# Anti-inflammatory activity of Wnt signaling in enteric nervous system: *in vitro* preliminary evidences in rat primary cultures

**DOI:** 10.1186/s12974-015-0248-1

**Published:** 2015-02-03

**Authors:** Rosa Di Liddo, Thomas Bertalot, Anne Schuster, Sandra Schrenk, Alessia Tasso, Ilenia Zanusso, Maria Teresa Conconi, Karl Herbert Schäfer

**Affiliations:** Department of Pharmaceutical and Pharmacological Sciences, University of Padova, Via Marzolo 5, 35131 Padova, Italy; Department of Biotechnology, University of Applied Sciences Kaiserslautern, Zweibrücken, Germany

**Keywords:** Wnt signaling, Frizzled 9, Wnt3a, LPS, Gut inflammation, Enteric nervous system

## Abstract

**Background:**

In the last years, Wnt signaling was demonstrated to regulate inflammatory processes. In particular, an increased expression of Wnts and Frizzled receptors was reported in inflammatory bowel disease (IBD) and ulcerative colitis to exert both anti- and pro-inflammatory functions regulating the intestinal activated nuclear factor κB (NF-кB), TNFa release, and *IL10* expression.

**Methods:**

To investigate the role of Wnt pathway in the response of the enteric nervous system (ENS) to inflammation, neurons and glial cells from rat myenteric plexus were treated with exogenous Wnt3a and/or LPS with or without supporting neurotrophic factors such as basic fibroblast growth factor (bFGF), epithelial growth factor (EGF), and glial cell-derived neurotrophic factor (GDNF). The immunophenotypical characterization by flow cytometry and the protein and gene expression analysis by qPCR and Western blotting were carried out.

**Results:**

Flow cytometry and immunofluorescence staining evidenced that enteric neurons coexpressed Frizzled 9 and toll-like receptor 4 (TLR4) while glial cells were immunoreactive to TLR4 and Wnt3a suggesting that canonical Wnt signaling is active in ENS.

Under *in vitro* LPS treatment, Western blot analysis demonstrated an active cross talk between canonical Wnt signaling and NF-кB pathway that is essential to negatively control enteric neuronal response to inflammatory stimuli. Upon costimulation with LPS and Wnt3a, a significant anti-inflammatory activity was detected by RT-PCR based on an increased *IL10* expression and a downregulation of pro-inflammatory cytokines *TNFa*, *IL1B*, and interleukin 6 (*IL6*). When the availability of neurotrophic factors in ENS cultures was abolished, a changed cell reactivity by Wnt signaling was observed at basal conditions and after LPS treatment.

**Conclusions:**

The results of this study suggested the existence of neuronal surveillance through FZD9 and Wnt3a in enteric myenteric plexus. Moreover, experimental evidences were provided to clarify the correlation among soluble trophic factors, Wnt signaling, and anti-inflammatory protection of ENS.

## Background

In the last two decades, enormous progress has been made in the characterization of soluble factors that regulate gut functionality at physiological and inflamed conditions. Among signaling molecules, Wnt family proteins (Wnts) are reported to play a pivotal role in the development of gut [1,2] balancing the homeostasis of intestine epithelium [3]. After the binding of Wnt ligands with the G protein-coupled receptor Frizzled (FZD) and single-span low-density lipoprotein receptor-related protein (LRP) [4,5], β-catenin is released from the large destruction “scaffolding” complex consisting of Axin, adenomatous polyposis coli (APC), casein kinase 1 (CK1), glycogen synthase kinase 3β (GSK3β) complex, and shuttles to the nucleus promoting the transcription of target genes [http://www.stanford.edu/~rnusse/wntwindow.html]. Moreover, under active inhibition induced by GSK3β or cross talked pathways [6-9], β-catenin is trapped at the plasma membrane or is tagged by phosphorylation for ubiquitin-mediated degradation [10].

Wnt ligands and Frizzled members have been largely studied in animal and human tissues [4,11]. Although the expression of Wnts, Frizzled, and Wnt-Frizzled binding specificities is well defined in epithelial and mesenchymal gut compartments [3], so far they have been poorly investigated in the enteric nervous system (ENS). The control of canonical Wnt pathway on intestinal tract has been first hypothesized observing β-catenin stabilization in familial and sporadic colon cancers [12]. Furthermore, significant evidences have demonstrated that Wnt ligands control the proliferation, differentiation, and self-renewal of intestinal crypt progenitor cells [13-15], in both paracrine and autocrine fashion [16]. As suggested by the increased expression of Wnts and Frizzled receptors in inflammatory bowel disease (IBD) and ulcerative colitis [17], Wnt signaling is involved in gut inflammation and exerts both anti- and pro-inflammatory functions. In particular, β-catenin has shown to i) negatively regulate intestinal NF-кB activity in bacterial-induced epithelial inflammation [18], ii) reduce TNFa release [19], and iii) induce the expression of *IL10* and TGFB [20] while the activation of pro-inflammatory mediators seems to be correlated to noncanonical Wnt signaling [21].

The course of Wnt FZD signaling is dictated by the specificity of Wnt-FZD interactions, which is in turn governed by both cell type and stage of development [16,22-24]. Expressed in the dorsal neural tube at the time of neural crest emigration [25], Wnt3a activates β-catenin target genes that are involved in several processes such as the positioning/maturation of Paneth cells and the proliferation and differentiation of colonic stem cell compartment [3,26,27] but also the maintenance and expansion of enteric neural crest progenitor cells [28]. As the release of Wnt3a increases in response to gut mucosa injury and inflammatory bowel diseases [29-31], the activation of canonical signaling pathway by Wnt3a could be hypothesized as an active mechanism in ENS.

Several studies have reported that Wnts preferentially activate the signaling pathway binding with defined members of FZD family according to a specific tissue distribution. Similarly to other Wnt ligands [32,33], a wide spectrum of FZD binding affinities for Wnt3a has been reported including FZD1 [19], FZD4, FZD5, and FZD8 [34]. Among recently investigated Frizzled receptors, FZD9 has been detected early during the development of the mouse nervous system [35] and this expression pattern is highly conserved like in chicken and zebrafish [36]. When FZD9 is activated by Wnt2, it leads to a Wnt/β-catenin signaling [37]. Although FZD9 is widely expressed on both multipotent neuroepithelial precursor cells and neural-restricted precursors [36,38], it was so far not yet described in the ENS compartment.

Besides Wnt proteins, glial cell-derived neurotrophic factor (GDNF), nerve growth factor (NGF), fibroblast growth factors (FGF), epithelial growth factor (EGF), leukemia inhibitory factor (LIF), interleukin 6 (IL6), and lipopolysaccharide (LPS) from gram-negative enteric bacteria [39-44] modulate strongly the ENS or gut function. In case congenital or acquired defects reduce the bioavailability of enteric neurotrophic factors, a compromised ENS development [45-48] and a dysfunction of the immune system [49] are observed to promote the inhibition of GSK3β activity [50,51] and enterocolitis [52], suggesting that a possible cross talk between Wnt/β-catenin pathway and immune response could be involved.

In experimentally LPS-induced colitis, an increased enteric glia activation [53] and neuronal cell death due to an excessive stimulation of excitatory transmitters [54] are reported to occur. The endotoxin LPS is an essential membrane component of luminal microflora bacteria that interacts specifically with enteric responsive cells through toll-like receptor 4 (TLR4) [55-57] to regulate intestinal homeostasis. In neuronal compartments, low-dose LPS can contribute to maintain neuronal survival [58] and differentiation through the activation of NF-кB, while higher doses result in excessive stimulation of proinflammatory cytokines [59] that leads to neuronal toxicity [60]. The involved intracellular signaling cascade includes the phosphorylation of mitogen-activated protein kinase (MAPK) and the migration of NF-κB into the nucleus [61]. Studies performed on enterocytes [62] showed that the response to LPS triggers the inactivation of GSK3β through the activation of phosphatidylinositol 3 kinase (PI3K)/Akt pathway [63,64]. There is increasing evidence that the critical activators of NF-кB pathway, IKKα, and IKKβ interact differently with β-catenin by regulating its protein levels and cellular localization [65]. In particular, unlike IKKβ that is predominantly cytoplasmic and activates NF-кB by phosphorylating IкBs under proinflammatory stimuli (TNFa, IL1, TLR agonists) [66], IKKα is detected in both nucleus and cytoplasm at resting state and inhibits β-catenin degradation mediated by Axin/APC/GSK3β, as well as it induces cyclin D1 expression that is a point of convergence between the Wnt/β-catenin and IкB pathways in mitogenic signaling [67]. As soon as the IKK complex is activated in response to mitogens, inflammation, apoptosis, immune response, or cancer, IKKα is enhanced to shuttle from cytoplasmic to nuclear compartment where it phosphorylates nuclear p65 within the transactivation domains and promotes the transcriptional activity of NF-кB target genes. While IKKβ-dependent pathway is essential for activation of innate immunity, IKKα-dependent pathway is more important for the regulation of adaptive immunity [66].

Based on these evidences, the present study was focused on the expression and activity of Wnt signaling components such as FZD9 and Wnt3a during the *ex vivo* growth of postnatal ENS cells. In particular, standard settings and starvation of growth factors were used to simulate physiological and pathological conditions, respectively, as the availability of soluble factors is already demonstrated to control the structural integrity and functionality of ENS. Taking into consideration that canonical Wnt signaling controls stem cell proliferation/differentiation [68-70] and inflammation [71], the immunophenotypical characterization of ENS subpopulations [72], all components of LPS/TLR4 signaling [56] and β-catenin modulatory activity on NF-кB were explored to better define the ENS response to inflammation [73-75].

## Methods

### Isolation of cells from rat enteric nervous system

ENS cells (ENSc) were isolated from Sprague Dawley rats, 3 days old as previously described [76] and under Italian and German ethic committee authorization (CEASA 43/2012). The isolated cells were either cultured in standard (SM) [76] and basal conditions (BM) [Neuronal Base P (PAA, Cölbe, Germany), 1% L-glutamine (Sigma-Aldrich, St. Louis, MO, USA), 1% penicillin/streptomycin (Invitrogen Life Technology, Carlsbad, CA, USA) for 7 days before morphological analysis by optical microscopy, immunophenotypical characterization by flow cytometry (FCM) and Wnt signaling study by gene expression, Western blotting (WB), protein complex immunoprecipitation (Co-IP), and immunofluorescence assay (IF) were performed.

### Immunophenotype characterization by FCM

The analysis was performed on ENSc at time of isolation (T0) and after 7-day culture (T7) using the primary antibodies reported in Table 1. In parallel, controls were stained using only corresponding secondary or isotypic antibodies. Data were acquired using FACSCanto II Flow cytometer (BD Biosciences, San Josè, CA, USA) and FACSDiva v6.1.3 software (BD Biosciences). The positive expression of each target marker was established using the overton subtraction tool of Summit 4.3 software (Beckman Coulter Inc, Brea, CA, USA).

**Table 1:** **Primary and secondary antibodies used for the immunophenotypical characterization of ENS cells**

***Primary antibodies***	***Manufacturing company***
Mouse anti-rat NG2 FITC	Santa Cruz Biotechnology, Inc
Goat anti-rat Nanog PE	BD Biosciences
Mouse anti-rat CD34 PECy7	BD Biosciences
Rabbit anti-rat Sox2	Millipore
Rabbit anti-rat Sox10	Santa Cruz Biotechnology, Inc
Rabbit anti-rat TLR4	Santa Cruz Biotechnology, Inc
Goat anti-rat Frizzled 9	Santa Cruz Biotechnology, Inc
Mouse anti-rat Nestin	Millipore
Mouse anti-rat GFAP	Millipore
Mouse anti-rat PAN neuronal	Millipore
Rabbit anti-rat p75	Millipore
*Secondary antibodies*	
Goat anti-mouse FITC	Santa Cruz Biotechnology, Inc
Goat anti-rabbit FITC	Santa Cruz Biotechnology, Inc
Bovine anti-goat FITC	Santa Cruz Biotechnology, Inc
Donkey anti-goat PE	Santa Cruz Biotechnology, Inc

### Immunofluorescence analysis

ENS cells cultured for 7 days (T7) in SM or BM were fixed with BD Cytofix solution (BD Biosciences), for 20 min, at 4°C. All samples were double stained with primary goat anti-rat Frizzled 9 or rabbit anti-rat TLR4 (Santa Cruz Biotechnology, Inc, Dallas, TX, USA) and, then, indirectly conjugated with PE and FITC secondary antibodies (Santa Cruz Biotechnology, Inc), respectively. In parallel, single staining with primary rabbit anti-rat Wnt3a (Cell Signaling Technology, Inc, Danvers, MA, USA) and FITC-conjugated secondary antibody was performed. As negative controls, specimens stained only with FITC- and PE-conjugated secondary antibodies were prepared. After mounting with Fluoro-Gel II solution containing DAPI (EMS, Hatfield, PA, USA), the samples were analyzed using a Leica TCS SP5 confocal microscope (Leica, Wetzlar, Germany).

### Formation of neurospheres under Wnt3a and LPS treatment

ENS cells were seeded at 16 × 10^3^/cm^2^ and cultured for 14 days with or w/o 5 μg/mL LPS (Sigma-Aldrich) or 20 ng/mL Wnt3a (R&D System, Minneapolis, MN, USA). After 1 (T1), 7 (T7), and 14 (T14) days, the number of neurospheres was counted using an Olympus CKX 41 microscope (Olympus, Hamburg, Germany) equipped with a Moticam 2500 and Motic Images Plus 2.0 software (Motic, Wetzlar, Germany). In parallel, the diameter of neurospheres was measured using the image processing software ImageJ.

### Wnt and LPS/TLR4 signaling pathway investigation

To investigate a possible interplay between Wnt and PI3K/Akt signaling pathways in ENSc, nuclear and cytoplasmic proteins were extracted from samples cultured in SM and BM and treated for 30 min, 1 h, and 2 h with 5 μg/mL LPS or 20 ng/mL Wnt3a. Proteins were obtained using NER PER Nuclear and Cytoplasmic Extraction Reagents kit and then quantified with BCA Protein Assay Reagent Kit (Thermo Fisher Scientific, Waltham, MA, USA) according to the manufacturer’s protocols. The separation of proteins was assessed by SDS/PAGE (Bio-Rad Laboratories, Inc, Hercules, CA, USA), and the immunoblot was carried out by overnight incubation, at 4°C, with primary rabbit anti-rat p(Ser9)-GSK3β (Cell Signaling Technology, Inc), mouse anti-rat p(Ser473)-Akt, β-catenin, p(Ser33)-β-catenin, NF-κB p50, and rabbit anti-rat NF-κB p65 (Santa Cruz Biotechnology, Inc) antibodies. The detection of target proteins was performed using peroxidase-conjugated goat anti-rabbit and goat anti-mouse secondary antibodies (Bio-Rad Laboratories, Inc). The development of immunoreactivity was enhanced by chemiluminescence substrate (ECL) (Sigma-Aldrich) and then visualized by VersaDoc Imaging System. The protein expression level was normalized to housekeeping protein GAPDH (Millipore) or lamin B (Santa Cruz Biotechnology, Inc) and quantified using the image processing software ImageJ. Data were reported as ratio within target protein and relative housekeeping protein expression.

### Co-IP: Wnt3a/Frizzled 9 (A) and NF-κB p65/β-catenin (B) binding assay

Total nuclear proteins were extracted from ENS cells cultured for 7 days (T7) using RIPA lysis buffer. (A) Recombinant Wnt3a protein (R&D System, Minneapolis, MN, USA) was added (200 ng/mL) to protein extracts and incubated overnight at 4°C. The immunoaffinity purification was performed as previously reported [77], using goat anti-rat Frizzled 9 (Santa Cruz Biotechnology, Inc) and rabbit anti-rat Wnt3a (Cell Signaling Technology, Inc) pre-immobilized onto Protein A Sepharose (Sigma-Aldrich). Western blot analysis was carried out using 4%–15% Mini PROTEAN® TGXTM Precast Gel (Bio-Rad Laboratories, Inc, Milan, Italy) and goat anti-rat Frizzled 9 (Santa Cruz Biotechnology, Inc) or rabbit anti-rat Wnt3a (Cell Signaling Technology, Inc). (B) For NF-κB p65/β-catenin binding assay, the immunoaffinity purification was performed using rabbit anti-rat NF-κB p65 or mouse anti-rat β-catenin (Santa Cruz Biotechnology, Inc), pre-immobilized onto Protein A Sepharose (Sigma-Aldrich). Both antibodies were used for Western blot analysis by 6.5% polyacrylamide gel (Bio-Rad Laboratories, Inc).

### Gene expression study: RT-PCR and qPCR

At 1 (T1) and 7 (T7) days from stimulation with Wnt3a and LPS, total cellular RNA was extracted using TRIzol® (Invitrogen Life Technology), quantified by measuring the absorbance at 260 nm and then stored at −80°C until use. Ten nanogram of RNA were reverse transcribed and amplified using Qiagen One Step RT-PCR Kit (Qiagen, Hilden, Germany) and an iCycler iQ™ (Bio-Rad Laboratories, Inc). Primer pairs for target and housekeeping genes were designed as reported in Table 2 and purchased from Invitrogen Life Technology. RT-PCR products were electrophoresed on a 2% agarose gel (Invitrogen Life Technology) stained with GelRed™ (Biotium, Inc, Hayward, CA, USA) and visualized using a UV transilluminator Gel Doc 2000 Gel Documentation System (Bio-Rad Laboratories, Inc). For genes reported in Table 3, the analysis was conducted by qPCR. In particular, the reverse transcription reaction was done with Thermoscript™ RT-PCR System kit (Invitrogen Life Technology) and iCycler iQ™ while the amplification reaction was carried out using Platinum® SYBR® Green qPCR SuperMix UDG kit (Invitrogen Life Technology) and a DNA Engine Opticon® Real Time Thermal Cycler (MJ Research, St. Bruno, QC, Canada). The amount of gene products was calculated using linear regression analysis from standard curves, demonstrating amplification efficiencies ranging from 95% to 100%. Data were reported as a fold increase of gene expression that is defined as the complementary DNA (cDNA) ratio between target gene and reference gene (*HPRT*) normalized to untreated sample. Statistical significance was calculated by Student’s *t*-test comparing to untreated samples: *p* value ≤0.05*, *p* value ≤0.01**; samples compared to LPS-treated cells: *p* value ≤0.01 (two black triangles).

**Table 2:** **Primer sequences for One-Step RT-PCR analysis**

**Gene**	**Abbreviations**	**Primer sequences**	**Accession**
Glial cell-derived neurotrophic factor	*GDNF*	F: CCAGAGAATTCCAGAGGGAAAG	NM_019139.1
		R: CTTCACAGGAACCGCTACAA	
Epidermal growth factor	*EGF*	F: GGGCTATCCCATCGGTAATAAG	NM_012842.1
		R: CAGCCTCCATTCCTGTGTAA	
Basic fibroblast growth factor	*BFGF*	F: AGAGGAGTTGTGTCCATCAAG	NM_019305.2
		R: CTCCAGGCGTTCAAAGAAGA	
Nerve growth factor	*NGF*	F: CAGTGTGTGGGTTGGAGATAA	NM_001277055.1
		R: GCATCCACTCTCTACAGGATTC	
Leukemia inhibitory factor	*LIF*	F: TGACGGATTTCCCACCTTTC	NM_022196.2
		R: CGTCTGTAGTCGCATTGAGTT	
Hypoxanthine-guanine phosphoribosyltransferase	*HPRT*	F: GCTGACCTGCTGGATTACAT	NM_012583.2
		R: CCCGTTGACTGGTCATTACA	

*F* forward, *R* reverse.

**Table 3:** **Primer sequences for qPCR analysis**

**Gene**	**Abbreviations**	**Primer sequences**	**Accession**
Toll-like receptor 4	*TLR4*	F: ATTGCTCAGACATGGCAGTTTC	NM_019178.1
		R: CACTCGAGGTAGGTGTTTCTGCTAA	
Wingless type MMTV integration site family, member 3A	*WNT3A*	F: TGCAAATGCCACGGACTATC	NM_001107005.2
		R: AGACTCTCGGTGTTTCTCTACC	
Frizzled 9	*FZD9*	F: TACCCAGAGCGCCCTATAAT	NM_153305.1
		R: CAAACCCTCCTGGATCACATAC	
Axis inhibition protein 2	*AXIN2*	F: ACCTATGCCTGTCTCCTCTAAC	NM_024355.1
		R: GTCCAGGGTATCCACACATTTC	
Myelocytomatosis proto-oncogene	*CMYC*	F: CTTGGAACGTCAGAGGAGAAA	NM_012603.2
		R: GCTTGAACGGACAGGATGTA	
Jun proto-oncogene	*CJUN*	F: GAAGCAGAGCATGACCTTGA	NM_021835.3
		R: CCATTGCTGGACTGGATGAT	
Interleukin-1β	*IL1B*	F: AGTGAGGAGAATGACCTGTTC	NM_031512.2
		R: CGAGATGCTGCTGTGAGATT	
Interleukin-6	*IL6*	F: GCCAGAGTCATTCAGAGCAATA	NM_26744.1
		R: GTTGGATGGTCTTGGTCCTTAG	
Interleukin-10	*IL10*	F: ATTGAACCACCCGGCATCTA	NM_012854
		R: CAACGAGGTTTTCCAAGGAG	
Tumor necrosis factor α	*TNFa*	F: GCAGATGGGCTGTACCTTATC	NM_012675.3
		R: GGCTGACTTTCTCCTGGTATG	
Hypoxanthine-guanine phosphoribosyltransferase	*HPRT*	F: GCTGACCTGCTGGATTACAT	NM_012583.2
		R: CCCGTTGACTGGTCATTACA	

*F* forward, *R* reverse.

## Results

### Growth factors modulate the expression of FZD9 and TLR4 positive cells

**Figure 1:**
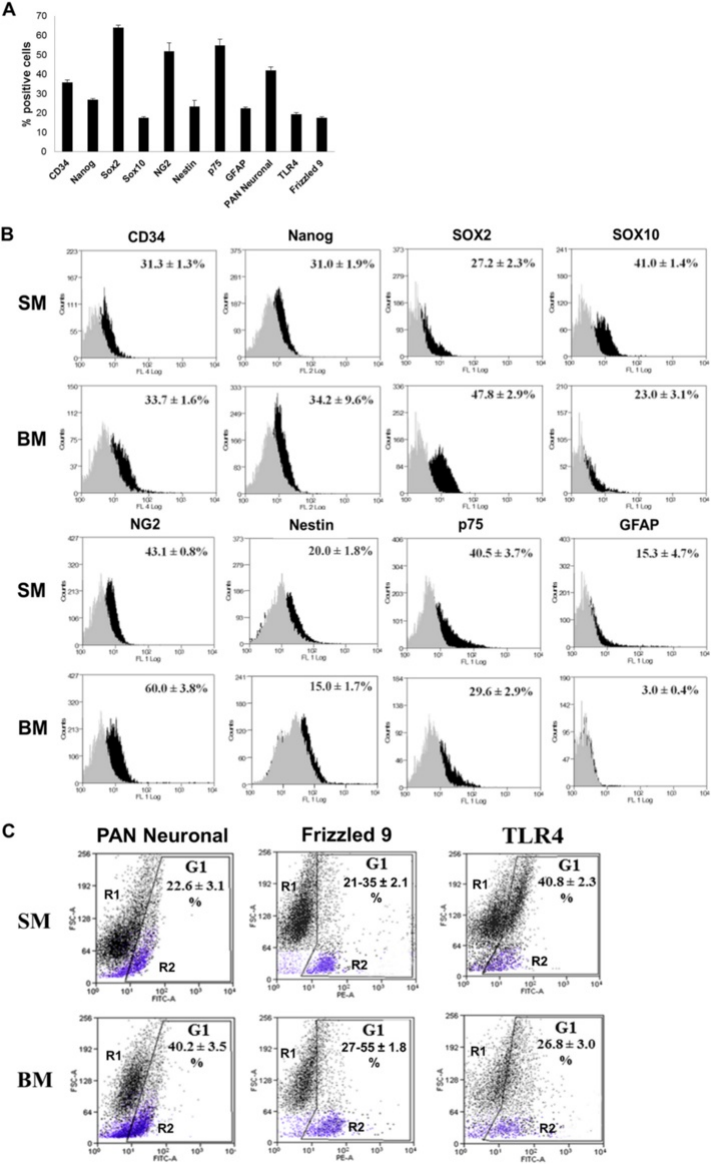
**FCM analysis of ENSc at T0 and T7. (A)** As reported by FCM analysis, freshly isolated ENSc (T0) resulted as a heterogeneous population including stem-like, progenitor, glial, and neuronal cells. For each experimental condition, 10^4^ cells were used for acquisition by FACSCanto II and data were expressed as percentage (%) of positive cells ± standard deviation (SD) where each marker was compared to control samples stained only with isotype or secondary control antibody. The positive expression was quantified using the overton subtraction tool of Summit 4.3 software (Beckman Coulter Inc). Under SM and BM culture conditions, the aspecific modulation of glial and neuronal differentiation was detected. **(B)** Immunophenotypical characterization by FCM of ENSc cultured for 7 days (T7). Data were expressed as percentage (%) of positives (black profile) ± SD for each marker compared to corresponding staining control (gray profile). **(C)** FCM analysis of PAN neuronal, Frizzled 9, and TLR4 expression. Data were reported as FSC vs fluorescent marker dot plot where R1 (blue colored) and R2 (black colored) subsets were defined. The positive expression of each target marker ± SD was discriminated in gate G1 defined with respect to staining control.

By flow cytometric analysis, freshly isolated ENSc showed a heterogeneous immunophenotype with a specific stemness pattern (Figure 1A), as suggested at T0 by the expression of Nanog (26.9 ± 0.6%), Sox2 (64.9 ± 1.4%), Sox10 (17.7 ± 0.4%), and p75 (55.0 ± 3.2%). Multidifferentiative potential of freshly isolated rat ENS cells was confirmed by the expression of nerve/glial antigen 2 (NG2) (51.9 ± 4.3%), a proteoglycan typically observed on the membrane of multipotent neural stem cells. Moreover, the presence of nestin (24.0 ± 3.3%) and CD34 (35.9 ± 1.2%) combined with the absence of cKit and CD44 (data not shown) was correlated to the presence of an immature cell population including neural precursors. The presence of differentiated glial and neuronal cells was revealed by the expression of GFAP (22.5 ± 0.6%) and PAN neuronal (42.1 ± 1.8%), respectively. The detection of Frizzled 9 (17.5 ± 3.0%) was indicative of constitutive activity of Wnt signaling while the responsiveness of ENS populations to LPS stimulus was suggested by the expression of TLR4 receptor (19.5 ± 0.7%). After 7 days of culture, a different expression level of stem cell markers was observed in samples treated under simulated physiological (SM) and reduced conditions (BM) (Figure 1B). As previously reported, Sox2 showed to be downregulated in cells maintained in SM (27.2 ± 2.3%) and in BM (47.8 ± 2.9%). In contrast, a significant increase of positivity for the pluripotency marker Nanog (31.0 ± 1.9% in SM; 34.2 ± 9.6% in BM), Sox10 (41.0 ± 1.4% in SM; 23.0 ± 3.1% in BM), and p75 (40.5 ± 3.7% in SM; 29.6 ± 2.9% in BM) was detected. Although unchanged in SM (15.3 ± 4.7%), a significant decrease of GFAP expression was detected in samples under basal conditions (3.0 ± 0.4%). No significant alterations of CD34 (31.3 ± 1.3% in SM; 33.7 ± 1.6% in BM) and nestin (20.0 ± 1.8% in SM; 15.0 ± 1.7% in BM) level expression were identified. Characterized by the expression of PAN neuronal antigen and lower FSC value in FCM dot plots (Figure 1C, blue-colored R2 subset), the neuronal subpopulation increased significantly in BM (40.2 ± 3.5%) with respect to SM-treated samples (22.6 ± 3.1%). Interestingly, the expression of Frizzled 9 (21–35 ± 2.1% in SM; 27–55 ± 1.8% in BM) was restricted in neuronal population (Figure 1C, blue-colored R2 subset). In contrast, a FCM biparametrical analysis (FSC vs TLR4) showed that TLR4 was distributed either in neurons and glial subpopulation (Figure 1C, black-colored R1 subset) with higher expression in SM (40.8 ± 2.3%) compared to BM (26.8 ± 3.0%).

### Frizzled 9 is coexpressed with TLR4 in enteric neurons

**Figure 2:**
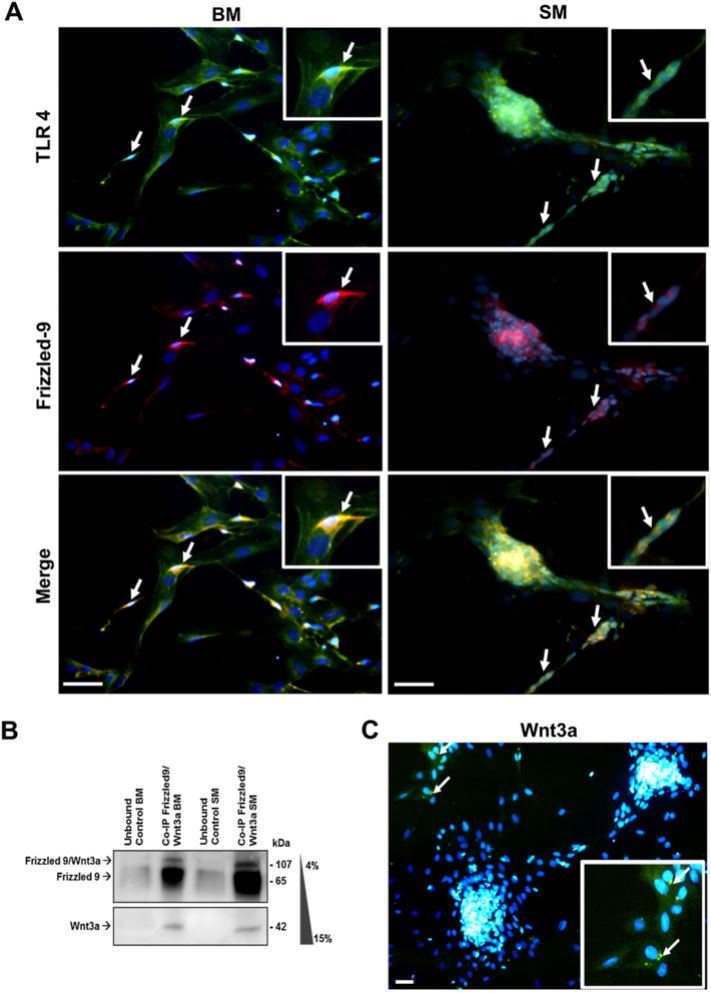
**IF analysis of TLR4 and Frizzled 9. (A)** Confocal microscopy analysis evidenced the coexpression of TLR4 and Frizzled 9 on neuronal cells (arrows) either in SM- than BM-treated samples. **(B)** Co-immunoprecipitation was performed on total protein extracts from ENSc cultured for 7 days in SM or BM. Immunoaffinity purification was carried out using goat anti-rat Frizzled 9 and rabbit anti-rat Wnt3a antibodies pre-immobilized onto Protein A Sepharose. Western blot analysis was assessed using 4%–15% gradient precast polyacrylamide gel. **(C)** By immunofluorescence assay, ENSc showed cytoplasmic vesicles immunoreactive (arrows) to rabbit anti-rat Wnt3a antibody. Bar: 15 μm.

By immunofluorescence staining, TLR4 and Frizzled 9 were confirmed to be coexpressed only in the neuronal subset (Figure 2A, arrows). When Co-IP was performed to investigate whether Frizzled 9 mediates canonical Wnt signaling, we evidenced a 107kDa band corresponding by molecular weight to Wnt3a/Frizzled 9 complex (Figure 2B) suggesting that Wnt3a could interact *in vivo* with Frizzled 9 and promote the activation of canonical Wnt signaling. Moreover, as cytoplasmic immunoreactivity for Wnt3a antibody was observed only in glial cells, a specific regulation of neuronal reactivity was hypothesized to be controlled by enteric glia by canonical Wnt (Figure 2C).

### Wnt3a and LPS enhance the proliferation of neurospheres

ENS-derived cells are usually cultured *in vitro* as aggregates known neurospheres that, including proliferating progenitors, neurons, and glial cells, mimic the *in vivo* niche of the gut. As growth factors and neurotrophic stimuli are demonstrated to be essential under *in vivo* settings for ENS functionality, they were used to study the regulation by Wnt signaling on the proliferation/differentiation of ENS cells under physiological conditions.

**Figure 3:**
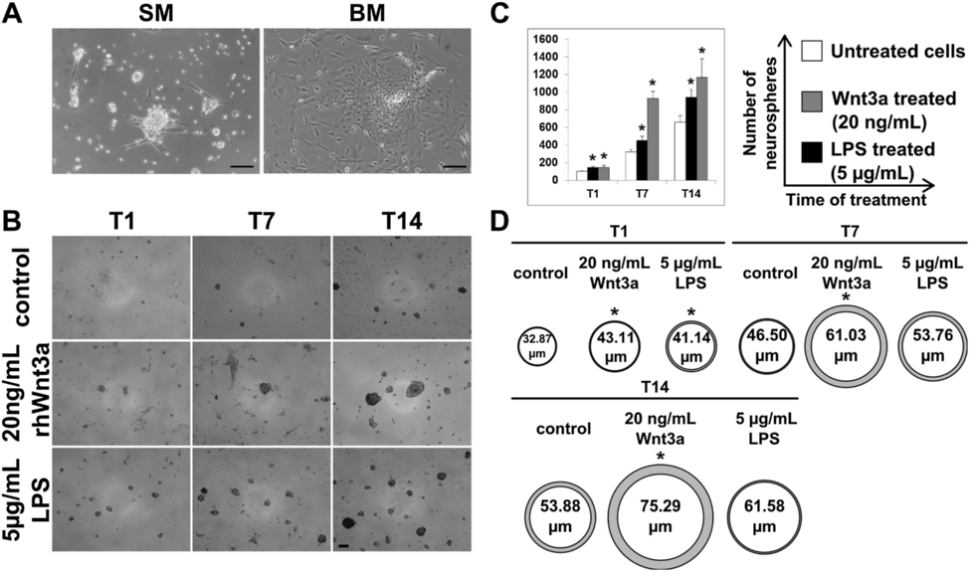
**ENSc and formation of neurospheres. (A)** After 1 day of culture, standard medium promoted the formation of neurospheres as evidenced by optical microscopy analysis. In contrast, several fibroblastoid colonies were observed in ENS cultures maintained in BM ENS medium. Bar: 100 μm. **(B)** Quantitative analysis of number and diameter of neurospheres obtained by LPS (5 μg/mL) or Wnt3a (20 ng/mL) stimulation. In parallel, the analysis was performed on LPS- and Wnt3a-untreated samples that were used as control. Light microscopic pictures reflected the increased number of neurospheres treated with LPS and Wnt3a after 1 (T1), 7 (T7), and 14 (T14) days in comparison to untreated cultures (control). **(C)** Data were quantified and normalized to controls (100%) (*n* = 3). **(D)** Diameter of neurospheres at T1, T7, and T14 was measured (*n* = 150) using ImageJ software. Statistical significance compared to control; *p* value ≤0.05*. Bar: 100 μm.

Typical neurospheres were detected by optical microscopy in ENS samples cultured in standard medium (Figure 3A). From 1 (T1) to 14 (T14) days of culture, LPS (5 μg/mL) and Wnt3a (20 ng/mL) demonstrated to induce in SM a significant (*p* ≤ 0.05%) increased number of neurospheres with higher extent in LPS-treated samples (Figure 3B,C). In parallel (Figure 3D), an enhanced proliferation rate was indirectly evaluated measuring the neurosphere diameter that resulted to be changed in control samples from 33 (T1) to 54 μm (T14) while in Wnt3a and LPS-treated samples increased from 43 (T1) to 75 μm (T14) and from 41 (T1) to 62 μm (T14), respectively. No neurosphere formation but several fibroblastoid colonies were observed in ENSc cultured in BM. These evidences suggested that the formation of neurospheres was strictly depending on growth factor stimulation.

### Canonical Wnt pathway is active in ENS cells and is modulated by growth factors

When the *in vivo* availability of soluble factors is restricted, the structural integrity of ENS is compromised with following inflammatory diseases. Using restricted culture conditions simulating a pathological disorder, we better defined the ENS response to inflammation.

**Figure 4:**
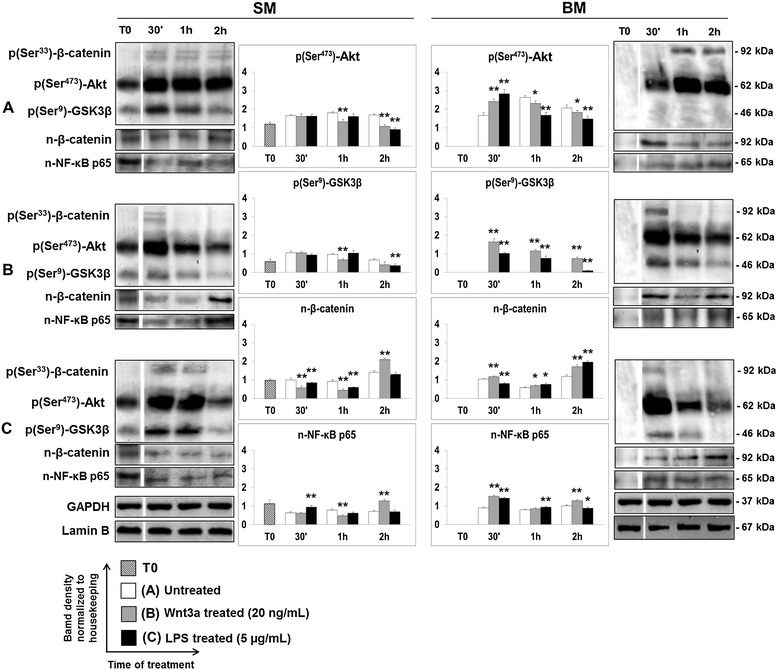
**WB analysis of Wnt/NF-κB interplay.** A cross talk between TLR4 and Wnt signaling occurred as demonstrated by WB analysis at T0, T30′, T1h, and T2h using specific antibodies for cytoplasmic p(Ser473)-Akt, p(Ser9)-GSK3β, p(Ser33)-β-catenin and nuclear β-catenin (n-β-catenin), and NF-κB p65 (n NF-κB p65) in unstimulated cells; **(A)** samples treated with Wnt3a (20 ng/mL) **(B)** and LPS (5 μg/mL) **(C)** in SM and BM medium. GAPDH and lamin B were considered as housekeeping proteins for the analysis of cytoplasmic and nuclear antigens. The quantification of protein expression levels was performed using ImageJ processing software. Data were reported as ratio within target protein and relative housekeeping protein expression. Statistical significance was calculated by Student’s *t*-test by comparing to control: *p* value ≤0.05*, *p* value ≤0.01**.

Both canonical pathway of Wnt signaling and LPS/TLR4 pathway play important roles in controlling the plasticity of ENS. Specific phosphorylation events control the active state of Akt [p(Ser473)-Akt], deactivate GSK3β [p(Ser9)-GSK3β], and tag β-catenin for proteosomal degradation [p(Ser33)-β-catenin]. In order to evaluate a cross talk between Wnt and NF-кB pathways, a protein expression analysis by WB was carried out to evaluate the phosphorylated state of Akt, GSK3β, β-catenin, and the nuclear translocation of both β-catenin (n-β-catenin) and NF-кB (n-NF-кB p65, n-NF-кB p50) (Figure 4). As phosphorylation events and cytoplasmic nucleus shuttling of transcription factors are very fast processes, WB analysis was performed using a time course ranging from 0 (T0) to 2 h (T2h) from start of stimulation. As shown in Figure 4A, an important modulation by supplemented growth factors was active on Wnt and LPS pathways involving the inactivation of GSK3β through its phosphorylation at Ser9 and the unmasking of cytoplasmic NF-кB, respectively. Moreover, the presence of nuclear β-catenin suggested that Wnt signal escaped from negative regulation in cytoplasmic compartment. Without any specific induction of Wnt or NF-кB signaling and under starvation of growth factors (BM), at T0 we observed that Akt was unphosphorylated, GSK3β was active, and the nuclear localization of β-catenin and NF-кB p65 was undetectable (Figure 4A). However, after 30 min from stimulation, pAkt was effective to contrast the negative regulation induced by GSK3β as demonstrated by the presence of β-catenin only in the nucleus together with NF-кB p65. The cytoplasmic level of β-catenin is controlled by Akt and GSK3β by phosphorylation tagging that, in our study, was interestingly increased when active Akt was more expressed (T1h, T2h) (Figure 4A). The lacking phosphorylation of GSK3β at Ser9 in BM-treated samples confirmed that PI3K stimulation by growth factors cross talks with Wnt signaling, as previously reported [6].

BM conditions showed to specifically discriminate the effects of Wnt3a and LPS on ENS cells. Similar expression profiles of β-catenin, Akt, and GSK3β were observed in samples treated with Wnt3a and LPS (Figure 4B,C) suggesting an interplay between Wnt and NF-κB pathways. In fact, in both cases, the activation of Wnt signal involved the phosphorylation of GSK3β at Ser9, the nuclear translocation of β-catenin and NF-κB p65, and the phosphorylation tagging of β-catenin only at early phase of stimulation (T30′).

### Wnt3a interferes with inflammatory ENS response

**Figure 5:**
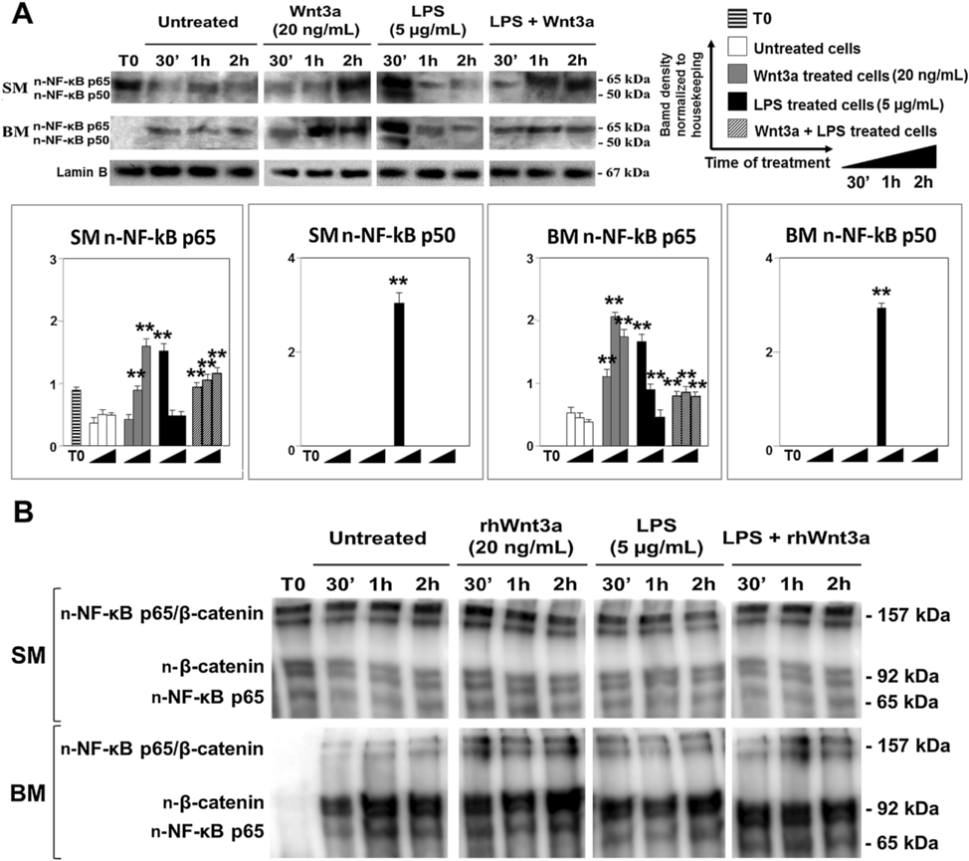
**WB and Co-IP of β-catenin and p65. (A)** Wnt3a interfered with NF-κB p50/p65-mediated inflammatory response as suggested by WB analysis of untreated Wnt3a (20 ng/mL) and/or LPS (5 μg/mL)-treated cells at T0, T30′, T1h, and T2h. The quantification of protein expression levels was performed using the image processing software ImageJ. Data were reported as ratio within target protein and relative lamin B housekeeping protein expression. Statistical significance was calculated by Student’s *t*-test comparing to control: p value ≤0.05*, *p* value ≤0.01**. **(B)** Co-IP assay confirmed a nuclear interplay between Wnt signaling and LPS NF-кB pathway, as previously reported [67]. Immunoaffinity purification was carried out using mouse anti-rat NF-κB p65 and mouse anti rat β-catenin antibodies pre-immobilized onto Protein A Sepharose.

As expected, NF-κB p50/p65 heterodimer was detected in the nuclear compartment at early phase of stimulation with LPS (T30') (Figure 5A). Interestingly, the presence of p65 subunit was observed in all other experimental conditions but not at T0 in BM suggesting that Wnt signaling interfered with canonical NF-κB pathway [78]. Moreover, we hypothesized that, due to alternative regulatory mechanisms, a possible specific stimulus-independent p65 translocation occurred [67,78]. In parallel, Co-IP assay demonstrated that NF-кB p65 was present in the nucleus both as free protein and β-catenin/p65 complex (Figure 5B). As β-catenin showed a similar conformation pattern, our data confirmed the hypothesis of a nuclear regulation of Wnt signaling on LPS/NF-кB pathway [67]. Unlikely BM-treated cultures, ENSc showed in SM an enhanced formation of the β-catenin/p65 complex with respect to free protein fraction.

### Exogenous Wnt3a exerts anti-inflammatory activity on ENSc

**Figure 6:**
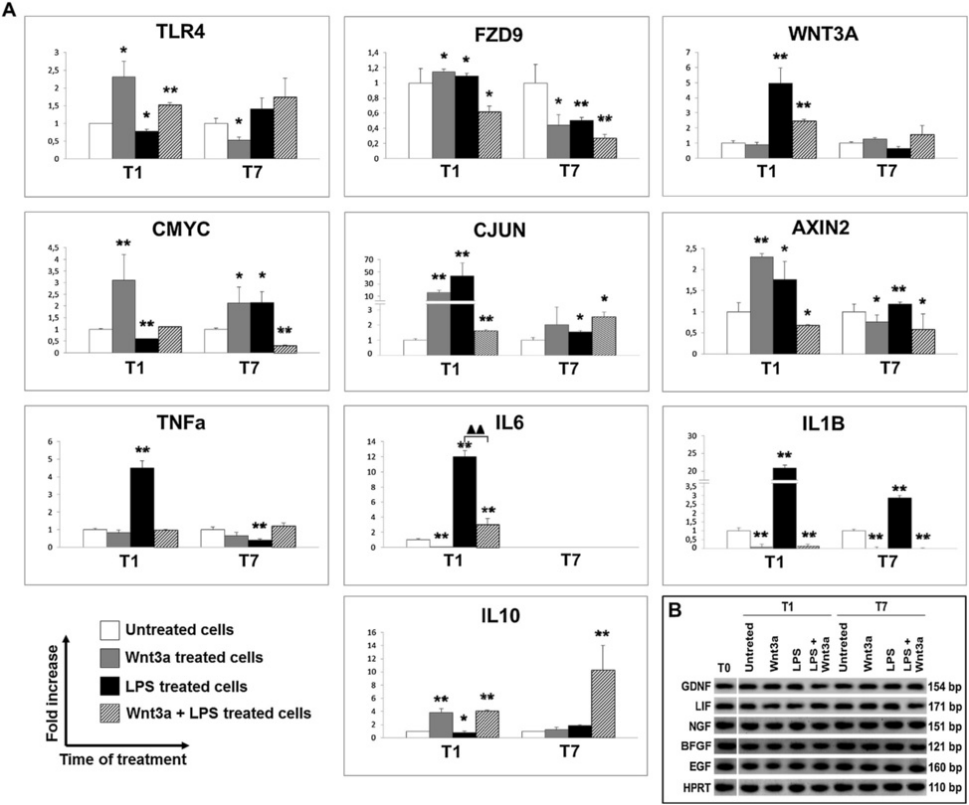
**Gene expression study on ENSc stimulated with Wnt3a and LPS. (A)** The anti-inflammatory effect was demonstrated to be exerted by Wnt3a using qPCR analysis and ENS cells treated with Wnt3a (20 ng/mL) and LPS (5 μg/mL). The analysis was focused on the expression of β-catenin target genes (*AXIN2*, *CMYC*, and *CJUN*), membrane receptors (*TLR4* and *FZD9*), Wnt3a ligand, and pro- and anti-inflammatory target genes (*IL1B*, *IL6*, *TNFa*, and *IL10*). The amount of gene products was calculated using linear regression analysis from standard curves, demonstrating the amplification efficiencies ranging from 90% to 100%. Data were reported as a fold increase of gene expression that is defined as the cDNA ratio between target gene and reference gene (*HPRT*) normalized to untreated sample. Statistical significance was calculated using Student’s *t*-test, comparing to untreated cells: *p* value ≤0.05*, *p* value ≤0.01**; samples compared to LPS-treated cells: *p* value ≤0.01 (two black triangles). **(B)** To detect the gene expression of typical ENS growth factors (GDNF, EGF, BFGF, NGF, and LIF), RT-PCR was performed using Qiagen One Step RT-PCR Kit on samples untreated (T0) and stimulated with Wnt3a (20 ng/mL) and LPS (5 μg/mL) for 1 (T1) and 7 (T7) days. RT-PCR products were electrophoresed on 2% agarose gel and stained by GelRed™. *HPRT* was used as housekeeping gene.

As suggested by RT-PCR (Figure 6B), a constitutive expression of *GDNF*, *EGF*, basic fibroblast growth factor (*BFGF*), *NGF*, and *LIF* was observed in all samples without any significant modulation by culture conditions and stimulation. These evidences highlighted that ENSc might modulate their reactivity to exogenous Wnt3a and LPS at longer time of culture (T7) through an endogenous production of growth factors. For this reason, in our opinion, the specific effect of Wnt3a and LPS could be really discriminated at early time of stimulation (T1). As *AXIN2*, *CJUN* and *CMYC* are reported as target genes of β-catenin transcriptional activity, their expression (*p* ≤ 0.01) at T1 (Figure 6A) was considered dependent on the specific activation of Wnt signaling in comparison to control. LPS treatment enhanced (*p* ≤ 0.05) the gene expression of *FZD9* and *WNT3A* (*p* ≤ 0.01) while decreased (*p* ≤ 0.05) the mRNA level of *TLR4*. Specific LPS-mediated response showed an increased expression (*p* ≤ 0.05) of *TNFa*, *IL6*, and *IL1B* genes while a reduced level (*p* ≤ 0.05) of *IL10* mRNA was observed. The costimulation with LPS and Wnt3a reversed the expression pattern of ENSc (*p* ≤ 0.01). Moreover, Wnt3a demonstrated to exert a negative control (*p* ≤ 0.01) on the gene expression of pro-inflammatory cytokines and to enhance ENS defense promoting an increased expression of anti-inflammatory *IL10* (*p* ≤ 0.01) and *FZD9* (*p* ≤ 0.01). Probably due to a feedback loop aimed to restore homeostatic conditions, *TLR4* expression was increased by Wnt3a (*p* ≤ 0.05) (Figure 6A).

## Discussion

When intestinal homeostasis [23] is disrupted by severe inflammation, pathologic changes of ENS neurons [79-81] and glial cells [82] occur, compromising gut motility and secretion [73,82,83]. In concentration-dependent manner [60], LPS contributes to ENS plasticity [84] and targets enteric neuronal and glial populations through TLR4 [85,86] promoting the secretion of pro- and anti-inflammatory effectors [87,88]. Recently, higher gene expression of Wnt3a and FZD9 has been demonstrated in IBD disorders in comparison to healthy patients [31] suggesting the involvement of canonical Wnt signaling into gut inflammatory response.

The current study identified rat FZD9 as a novel marker associated with rat myenteric plexus and its ability to respond to Wnt3a ligand activating β-catenin signaling. It is known that in embryonic [89] and postnatal gut [90], the components of Wnt signaling are expressed in complex spatial and temporal patterns according to specific activities. Previous studies on Frizzled 9 in human and mouse have shown that it is highly expressed in the brain [21,91] and in neural precursor cells during developing nervous system [36,92]. Moreover, FZD9 protein has been localized at growth cones of regenerating adult spiral ganglion neurons [93] suggesting to be involved in neuronal response to damage.

As previously reported [71], the cultures of dissociated postnatal gut give rise to multipotent ENS progenitors generating *in vitro* neurons and glia. Based on FSC morphological discrimination and positive reactivity to anti-PAN neuronal marker by FCM analysis, the expression of FZD9 was demonstrated to characterize the neuronal compartment and to be preserved under BM and SM culture conditions.

During ENS development, there is a critical balance among migration, survival, proliferation, and differentiation of neural crest cells (NCCs). A fine control of these processes guarantees that a sufficient number of NCCs enter the foregut at the correct time while proliferation and differentiation are maintained at a balanced level at migration wavefront. It is known that the microenvironment plays a pivotal role to regulate the extent of ENS formation influencing on NCC number and gut colonization. Although neurotrophic factors are demonstrated to be essential for gut formation, the biological processes controlled by these factors are not yet completely elucidated [94,95]. In both BM and SM, a stem cell subset including enteric nervous stem cells (ENSCs) [96,97] is maintained during *in vitro* culturing. Numerous evidences suggest that germinal niches are adjacent to myenteric ganglia, and myenteric plexus-derived preparations are enriched of ENSCs. Using genetic fate mapping of Sox10, a neural crest cell marker [98], it has demonstrated that in postnatal gut, Sox10 positive cells can act as neuroprogenitor cells and regenerate neurons after damage. CD34 has been demonstrated to be expressed in hematopoietic stem cells [99,100], putative endothelial cells [101], and mesenchymal precursor cells [102]. In the gut, CD34 expression has been reported either in interstitial Cajal cells together with cKit, CD44 [103], and ENSC [104] that, likely committed lineage cells [71,105-108], are commonly characterized by p75 [104,105] and Sox2 [106,109]. In our study, as previously shown by Hagl et al. [47], the expression of Nanog confirmed the existence of stem cells with high differentiative potentiality and the absence of cKit and CD44 led to consider CD34 as associated to an immature cell population rather than interstitial Cajal cells.

As suggested by the expression level of Sox10, p75, and GFAP, glial cells were stimulated under standard culture conditions while neuronal committed cells expressing Sox2, NG2 [110,111], and PAN neuronal marker were *in vitro* maintained and numerically increased when deprived of bFGF, EGF, GDNF, and NGF. These evidences suggested that a diminished availability of neurotrophic factors fostered neuronal commitment of progenitors but negatively controlled the cell differentiation process as shown by lacking neurosphere formation. In contrast, under standard culture conditions, glial differentiation was promoted more than neuronal and neurospheres were regularly formed.

Under Wnt3a (20 ng/mL) and LPS (5 μg/mL) stimulation, neurospheres were demonstrated to increase in number and size. After 1 day of culture, the neurosphere diameter was 10 μm bigger in LPS- and Wnt3a-treated samples than that observed in control. At T7, the number of neurospheres was doubled in LPS-treated samples compared to that detected under Wnt3a stimulation. Interestingly, a larger diameter of neurospheres was induced by Wnt3a with respect to LPS. The different proliferation effect could be indicative of a protective mechanism that, through the stimulation of immature cells but by the activation of different pathways, LPS [44] and Wnt3a [112] guaranteed a neural renewal.

Although FZD9 in human has been shown to bind Drosophila Wingless [91], the first evidence of its ability to activate Wnt/β-catenin signaling through the binding of Wnt2 ligand was obtained from Karasawa et al. [37] by an *in vitro* rat model.

The interaction between Wnts and Frizzled receptors is specific and differently involved in physiological and pathological conditions [3,113]. In our case, the demonstrated ability of FZD9 to bind Wnt3a and the presence of Wnt3a in glial cells provided the evidence of a possible protective role exerted by glia on enteric neurons through the activation of Wnt canonical pathway at physiological state or pathological conditions [114].

Wnt1 and Wnt3a are coexpressed at the dorsal midline of the developing neural tube and control the midbrain patterning and the formation of the paraxial mesoderm, respectively. When a deficiency of both Wnt1 and Wnt3a is observed, the development of neural crest derivatives is compromised suggesting that local Wnt signaling regulates the expansion of dorsal neural precursors. Despite the high number of mammalian Wnt and Frizzled members, a poor characterization of Wnt Frizzled binding specificities is reported.

In particular, it is known that canonical Wnt signaling controls crypt progenitor gene expression pattern [115]. Recently, several studies have implied the involvement of canonical Wnt Frizzled signaling in inflammatory bowel disease (IBD) [21,31], as a significant increased expression of Wnt3a and Frizzled receptors are observed [114]. In the current study, the nuclear translocation of β-catenin in rat ENS cells under stimulation with exogenous Wnt3a suggested that canonical Wnt pathway is active in postnatal myenteric plexus and Frizzled 9 could be involved as a specific ligand. The transcriptional regulation of FZD9 after the treatment with Wnt3a suggested that it might play a role in the context of inflammation. Only little is known about the expression and function of FZD9 in postnatal ENS. Among frizzled receptors, we demonstrated that FZD9 protein was significantly expressed on freshly extracted ENS cells and maintained in *ex vivo* cultured neuronal population. As previously reported [94], the regulation by Wnt signaling could be interpreted as protective and might be involved in the ENS defense. Activated Wnt3a/β-catenin signaling pathway is known to stimulate intestinal epithelial repair after wounding [116] and neural restricted precursor cell populations [117]. In our study, the binding specificity of Frizzled 9 with Wnt3a was demonstrated in ENS compartment and suggested the involvement of FZD9 in neuronal response to inflammation in light of the evidence that a significant increase of Wnt3a and FZD9 is observed in inflammatory bowel diseases [31]. The expression of TLR4 by the enteric neural cells highlighted the presence of a neural surveillance network that activates a cell-specific response against bacterial agents when the epithelial barrier permeability is altered [86]. In the present study, the expression of both TLR4 and Frizzled 9 was detected in the neuronal population, while glial cells were characterized only by TLR4. Our evidences suggested that a neuronal subset was responsive to inflammatory agents through TLR-based mechanism and was regulated by Frizzled 9-mediated canonical Wnt signaling.

The cross regulation of Wnt and NF-кB signaling pathways has been shown to modify the biological effects of gene expression during development, immune function, inflammation, and carcinogenesis. A wide spectrum of phosphorylation/acetylation events and associating proteins is demonstrated to modulate the activation or inhibition of β-catenin, NF-кB, or their binding activity to DNA [67], independently on specific stimuli. In the last two decades, a lot of experimental data have reported that the interplay between Wnt signaling and NF-кB is expressed at different levels and is subject to variations in dependence of PI3K/Akt pathway, cell type specificity, and physiological/pathological conditions [118].

In the present work, we have demonstrated that canonical Wnt signaling is active in ENS and cross talks with NF-кB modulating the response to LPS. Phosphorylation events at serine 473 activate Akt and lock it at active conformation [119]. In both standard and basal medium, the presence of nuclear β-catenin and NF-кB was observed in the absence of exogenous stimulation with Wnt3a and LPS and thus was correlated with active p(Ser473)-Akt.

As p(Ser9)-GSK3β was detected only in standard medium and p(Ser33)-β-catenin started to be expressed at T1h, we hypothesized that Akt controls phosphorylation events in resting cells promoting directly or indirectly both activation and degradation of β-catenin, besides nuclear translocation of NF-кB p65. It was demonstrated that Akt phosphorylates β-catenin at Ser552 leading first to its dissociation from cell-cell contacts and then to the increase of its binding to 14 3 3 proteins and its transcriptional activity [120]. As GSK3β is active in resting cells and is inactivated through serine 9 phosphorylation induced by mitogens, growth factors, and several kinases, including Akt [121], we hypothesized that endogenous factors could contribute to regulated activation of Akt and control the stabilization of β-catenin by IKKα [65,67]. Wnt- and FGF-dependent PI3K Akt signaling pathways cross talk during a variety of cellular processes including neurogenesis [122] and both lead to GSK3β downregulation although depending on different phosphorylation events. In contrast to FGF signaling, the canonical Wnt is reported to be independent on Ser 9 phosphorylation. In the present work, we demonstrated that Wnt3a and LPS cross talk at the level of GSK3β and deactivate it by Ser9 phosphorylation, even if other phosphorylation events are not excluded because our *in vitro* model is not based on the inhibition of PI3K/Akt signaling pathway but only on the deprivation of main ENS trophic factors. This activity is hypothesized to be specifically dependent on the activation of Wnt and NF-кB signaling, probably modulated by GSK3β but not depending on exogenous growth factors such as bFGF and EGF as the evidences were taken from basal medium-treated samples. The concomitant presence of p(Ser33)-β-catenin and p(Ser9) GSK3β at early phase of Wnt3a and LPS stimulation suggested that alternative mechanisms of phosphorylation might occur promoting the negative control of β-catenin and thus its modulation. As the protein expression profile detected in standard and basal medium-treated samples showed a stronger accumulation of β-catenin at late phase of Wnt3a stimulation, GSK3β downregulation by simultaneous treatment with Wnt and growth factors is hypothesized to involve GSK3β pools differently modulated by Akt.

The critical role of Akt signaling for neuroprotection against deprivation of growth factors [123], oxidative stress [124], and ischemic injury [125] is evidenced from several *in vitro* studies on neuronal cell lines or primary cultures. Phosphorylation of Akt through phosphatidylinositol 3 kinase is essential to promote ENS precursor survival [8]. Akt level expression was correlated with a constitutive activation of a neuroprotective mechanism as previously reported by Humbert et al. [126]. Interestingly, in basal medium-treated samples, LPS response evidenced a superior expression level of nuclear β-catenin than that observed in ENS cells cultured in standard medium probably due to an interaction of Akt with GSK3β [127]. As BM-based *in vitro* model could simulate pathological conditions, wherein the limited or absent supply of growth factors promotes gut inflammation, the higher nuclear translocation of β-catenin could be interpreted as a negative regulation of NF-кB p65 transcriptional activity.

**Figure 7:**
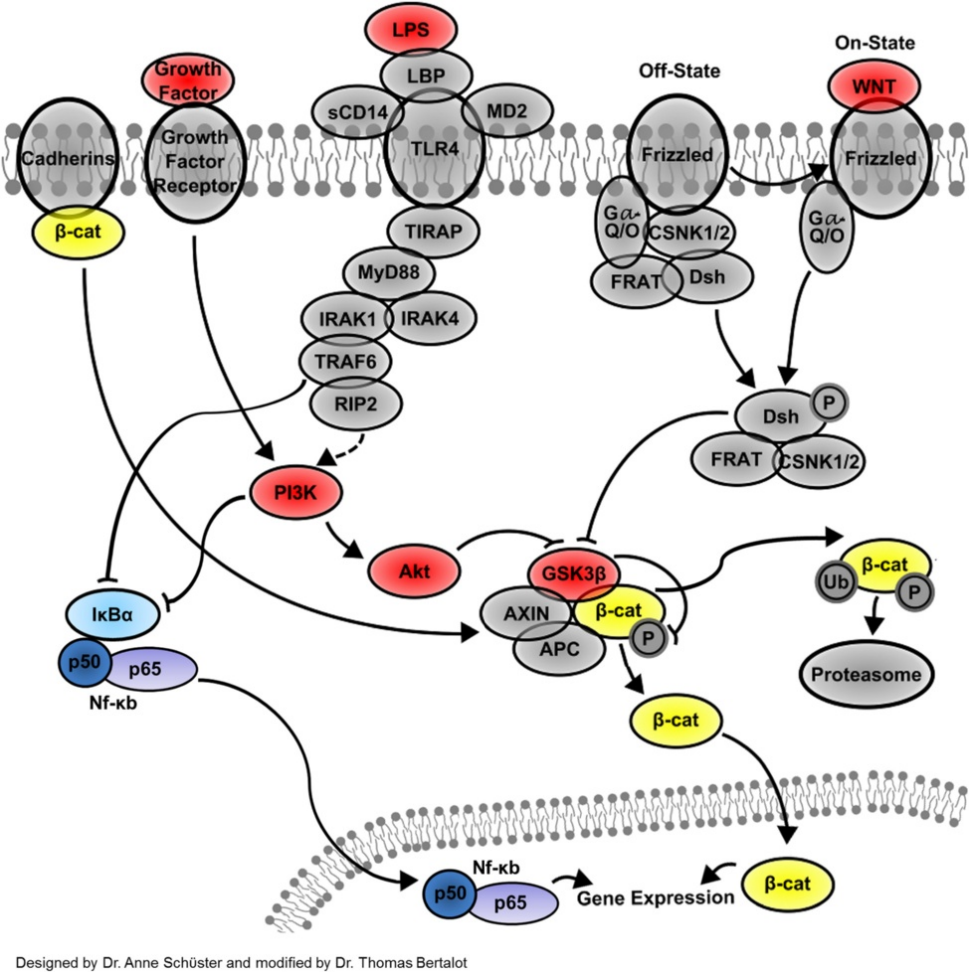
**The interplay model of Wnt/β-catenin, LPS/TLR4, and growth factors in ENS.** After the interaction of Wnt ligands with a G protein-coupled receptor (Frizzled), GSK3β is inhibited and β-catenin is released from a “scaffolding” complex consisting of Axin, adenomatous polyposis coli (APC), casein kinase 1 (CK1), and glycogen synthase kinase 3β (GSK3β). Consequently, the stabilized β-catenin shuttles to the nucleus and the transcription process of its target genes is promoted. LPS-mediated activation of TLR4 pathway or growth factor signaling involves phosphatidylinositol 3 kinase (PI3K) pathway. PI3K inactivates GSK3β through Akt, and nuclear accumulation of β-catenin occurs. In steady conditions, β-catenin is trapped at the plasma membrane by cadherins or is tagged by phosphorylation for ubiquitin-mediated degradation. In parallel, PI3K and TRAF6 inhibit IκBα and the activated heterodimer NF-κB p50/p65 translocates to the nucleus for promoting the specific gene expression.

As GSK3β has been shown to regulate NF-кB at the level of transcriptional complex [128] and β-catenin is a major substrate of GSK3β, it is hypothesized that β-catenin might serve as a mediator for the cross regulation between Wnt and NF-кB signaling pathways at different levels and cellular compartments (Figure 7). The binding of β-catenin and NF-кB on specific regions of DNA is suggested to be fine regulated, as the activation and nuclear translocation of both transcription factors might be promoted without any specific stimulated signaling pathway, but only due to a cascade of phosphorylation events.

In the present study, using Co-IP technique, we demonstrated that nuclear β-catenin and NF-кB p65 could complex together or are present as free proteins in resting ENS cells. The β-catenin/p65 complex and the free proteins resulted to be differently expressed in dependence on experimental conditions. It is already reported that β-catenin can physically interact with p65 and p50 subunits to inhibit the binding of NF-кB to DNA in IкBα-independent manner and in association with additional cellular factors [78]. Interestingly, suppressed NF-кB activity and target gene expression were observed in cells expressing high level of β-catenin [78]. Hwang et al. [112] have proposed a model of combinatorial binding of p65 and β-catenin showing that the specific transcriptional activity of β-catenin is regulated by protein-protein interaction with p65 at transcriptional level of their target genes. As the promoters of Wnt signaling target genes contain NF-кB binding elements, in the absence of specific stimulation, a constitutive binding of β-catenin/p65 complex to NF-кB sites is suggested to block β-catenin transcriptional activity. After Wnt signaling activation, the nuclear accumulation of β-catenin leads to the displacement of p65 from gene promoters and to the binding of β-catenin TCF transcriptional complex to DNA. Under LPS activation, IKKα-mediated phosphorylation of nuclear p65 subunit within the transactivation domains promotes the transient transcriptional activity of NF-кB target genes [67].

The major expression of β-catenin/NF-кB p65 complex in SM-treated samples was correlated to the greater accumulation of nuclear β-catenin and NF-кB p65 deriving from aspecific stimulation of NF-kB (or alternative NF-кB pathway) and Wnt signaling pathway exerted by trophic factors [127]. Only the stimulation with LPS resulted into the activation of canonical NF-кB pathway, as demonstrated by the nuclear presence of NF-кB p50/p65. This evidence confirmed that a large diversity of signals might converge on degradation of IкBs, the known proteins that physically mask the nuclear localization signal (NLS) of NF-кB p65. However, the DNA binding activity of NF-кB to IкB sites, among a large excess of potential binding sites, is specifically controlled by NF-кB subunits, associated proteins, and binding to nuclear chromatin.

The specific responsiveness of ENS cells to Wnt3a or LPS stimulation was confirmed evaluating the transcriptional activity of nuclear β-catenin and p65 subunit in samples cultured in basal medium. Both canonical Wnt signaling and NF-кB pathway are expressed in ENS and control gut homeostasis [129]. As the FZD9 expression was observed only in neuronal subpopulation while that of TLR4 resulted on both neurons and glial cells, we asked us which reciprocal regulation occurs between canonical Wnt pathway and NF-кB signaling in ENS neurons at physiological and inflamed conditions. The high level of GSK3β expression in brain tissue is likely due to its essential role in neuronal signaling. In neuronal cells, it is required for dendrite extension and synapse formation in newborns. Dysregulation of GSK3β expression leads to many pathological conditions, including neuronal dysfunction, Alzheimer’s disease [130], and Parkinson’s disease [131]. Highly expressed in colorectal cancer [132,133], it has been shown to participate in NF-кB-mediated cell survival in pancreatic cancer [134]. In cell culture studies, apoptosis is attenuated or fully abrogated by the inhibition of GSK3β in primary neurons [135] and neuronal cell lines [136]. The Wnt pathway is involved in regulation of gut homeostasis. Under intestinal inflammation, the expression level of Wnt3a significantly increases [31] and neuronal apoptosis is observed [60].

Schaale et al. [137] described a regulatory role of Wnt signaling in inflammatory processes and reported that exogenous Wnt3a mediates anti-inflammatory effects in macrophages promoting the suppression of proinflammatory cytokines. Moreover, Li et al. demonstrated that Wnt signaling contributes to Alzheimer’s disease-related neurodegeneration by regulating neuroinflammation [138] and plays a crucial role in the expression of proinflammatory cytokines in the central nervous system [139]. In our study, LPS treatment enhanced the gene expression of FZD9 and Wnt3a but reduced the level of *TLR4* mRNA, suggesting a possible negative feedback scenario in which a positive regulation of Wnt pathway could be exerted by NF-кB signaling to restore homeostatic conditions. This hypothesis was further confirmed evaluating that the specific LPS-mediated inflammatory response was reversed in the presence of Wnt3a as demonstrated by a reduced expression of *TNFa*, *IL6*, and *IL1B* genes and, interestingly, an increased level of *IL10* mRNA. As it is reported that LPS-treated enteric neurons respond stimulating the secretion of inflammatory cytokines such as IL6, TNFa [129] to activate glial cells that, in turn, release IL1B, the inhibitory activity exerted by Wnt3a could be interpreted as effective to control glial activation.

Taken together these considerations, we concluded that Wnt3a exerts a negative control of NF-кB transcriptional activity in rat myenteric plexus and contributes to ENS defense by the stimulation of *IL10*, *TLR4*, and *FZD9* expression. In turn, LPS balances the inflammatory response stimulating Wnt signaling and enhancing the transcription level of *FZD9* and Wnt3a.

## Conclusions

The results of this study suggested the existence of neuronal surveillance through FZD9 and Wnt3a in enteric myenteric plexus. As detected in freshly isolated ENS cells, FZD9 was hypothesized to be expressed in constitutive manner for some homeostatic activities. Although the total number of FZD9 positive cells cultured under standard and basal conditions did not significantly change, the expression level of FZD9 was upregulated in response to GDNF, bFGF, and NGF, suggesting a possible involvement of Wnt signaling in neuronal and glial differentiation. Interestingly, under *in vitro*-simulated inflammation, Wnt signaling was demonstrated to exert an anti-inflammatory activity to negatively control NF-кB pathway.
